# Afterword: Police, policing, and HIV: new partnerships and paradigms

**DOI:** 10.1186/1477-7517-9-32

**Published:** 2012-07-09

**Authors:** Chris Beyrer

**Affiliations:** 1 The Center for Public Health and Human Rights, Johns Hopkins University, Baltimore, MD, 21205, USA

## 

The important work in this volume tells us some powerful truths. It has long been clear that law enforcement has critical roles to play in protecting the health of the public, and that law enforcement entities have legitimate concerns of public safety, security, and protection. That these roles have led to a broad array of responses to substance use and substance users, which have ranged from harmful to helpful, has also been clear. But in the research and policy pieces brought together here by Nick Crofts and Nicholas Thompson and colleagues, a new paradigm emerges with the potential to have real impacts on key shared goals. Harm reductionists seek to see declines in the health and social consequences of substance use, including reductions in new HIV infections that will be so key to the trajectory of HIV epidemics in much of the world. Law enforcement seeks to see declines in crime, in insecurity, and increases in public safety. Both groups, and all of society more broadly, need to seek declines in unnecessary detention, incarceration, and the destruction of young lives which the use of detention for substance use has wrought. The new paradigm suggests that these goals are not inherently antagonistic—and indeed, are powerfully *synergistic.* How?

By re-envisioning the role of law enforcement as supporting harm reduction, evidence-based substance use treatment, and the protections of human rights for all citizens, the goals of disease prevention and public safety can both be met. Harm reduction is an effective outreach tool for prevention, for public safety, and for accessing those who want, and need, access to drug treatment services. Partnering with law enforcement in new strategic alliances can empower public health authorities, and allow the voices and concerns of substance users to be heard in debates relevant to their lives and needs. And, as Professor Crofts has shown, police to police dialogues can be steps in the process of moving from aggressive resistance to harm reduction, to an embrace of the principles of engagement, health-centered approaches, and pragmatism.

What remains to be done? As the reports here demonstrate, the ongoing use of detention, particularly novel forms of administrative detention without trial for substance users in Southeast Asia and in China, have emerged as real threats to the health and wellbeing of people who use substances or who have been detained for their putative use. These facilities continue to expand in too many countries, and continue to profit from the unpaid labor of detainees. This approach to substance use violates public health principles and human rights, but also undermines the role of law enforcement, creating parallel systems under uncertain legal grounds and often outside the scrutiny of courts and legal systems. This is bad public policy, bad public health, and threatens to undermine the impressive advances in policing practice documented here.

We can do better. And the evidence now suggests that many states and countries are doing better. The theme of the 19^th^ International AIDS Conference in July of 2012 is “Turning the Tide Together.” The theme attempts to capture the new optimism that an “AIDS Free Generation” is possible with the impressive new tools and policies we now have to fight HIV. But this optimism is tempered by the very real concern that HIV continues to spread in some key populations, including people who inject drugs. Yet this is one population where the evidence is the most abundant that HIV spread can be almost entirely prevented with an affordable package of services, including harm reduction, effective drug treatment, and access to anti-viral therapy for those living with the virus. This is one fight we know how to win. But we cannot win it without the engagement and support of law enforcement as partners in the HIV effort. With the new paradigm presented here this fight can be won, and an AIDS free generation just might be possible. What an achievement that would be for all who are working on these issues, public health, affected communities, and law enforcement. That’s the “together” part of the AIDS 2012 theme, and you have in your hands part of the blueprint for the way forward.

